# Gain‐of‐function mutation Met136Val in *SCN8A* may not be a common cause of trigeminal neuralgia

**DOI:** 10.1002/mgg3.1587

**Published:** 2021-01-11

**Authors:** Raymond F. Sekula, Kathleen Deeley, Hayley Denwood, Alexandre R. Vieira

**Affiliations:** ^1^ Department of Neurosurgery School of Medicine University of Pittsburgh Pittsburgh PA USA; ^2^ Department of Oral Biology School of Dental Medicine University of Pittsburgh Pittsburgh PA USA

## Abstract

**Background:**

The Met136Val mutation in *SCN8A* was described in a case of trigeminal neuralgia but no frequency among affected individuals was provided.

**Methods:**

Direct sequencing of 123 individuals diagnosed with classic trigeminal neuralgia was performed aimed to detect the Met136Val change.

**Results:**

No cases of classical trigeminal neuralgia studied had the Met136Val mutation in *SCN8A*.

**Conclusion:**

Met136Val mutation in *SCN8A* is not a frequent cause of classical trigeminal neuralgia.

## Graphical abstract

This paper provides evidence that the SCN8A Met136Val is not a common cause of trigeminal neuralgia.

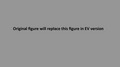

Classical trigeminal neuralgia (cTN, OMIM accession number 190400) is a neuropathic pain disorder marked by evoked and spontaneous attacks in the distribution of the trigeminal nerve, and the disorder is further associated with the periods of complete remission and subsequent recurrence in most patients. The pain of cTN is considered to be among the most debilitating types of pain (Cruccu et al., 2010). Neurovascular compression of the trigeminal nerve has been accepted as the cause of cTN in a majority of patients by the International Headache Society. The condition is usually sporadic, although a recent publication suggests that familial occurrence may be more common than previously considered (Di Stefano et al., 2020). A mutation in *SCN8A* (OMIM accession number 600702. GenBank AH007414.2), a sodium channel gene that codes for the Na_v_1.6 protein, was reported in a 64‐year‐old white female that presented with classical trigeminal neuralgia. The Met136Val change (NM_014191.4:c.406A>G) produced a number of detectable changes in neurotransmission. It was possible to detect a significant increase in peak transient and resurgent currents of Na_v_1.6. The mutation also reduced the threshold for action potential in trigeminal ganglia neurons and enhanced the neuronal evoked response and the fraction of neurons that fire at a higher rate than those expressing wild‐type channels (Tanaka et al., 2016). Pathogenic variants in *SCN8A* are also known to cause SCN8A‐related epileptic encephalopathy (Hammer et al., 2016). Since it was not clear from the original report how frequent the Met136Val variant in *SCN8A* is among individuals with trigeminal neuralgia, we tested the hypothesis that the mutation *SCN8A* Met136Val is an infrequent finding in individuals with trigeminal neuralgia.

One hundred and twenty‐three individuals diagnosed with trigeminal neuralgia were recruited since January 2016 to be part of our Orofacial Pain Registry and Sample Repository project (IRB approval # 15110027). They were 81 females, 110 non‐Hispanic Whites, and all adults at least 45 years of age. These individuals provided written informed consent and a biological sample (unstimulated saliva) and are from the most part from the western Pennsylvania region. All cases were diagnosed by the same professional (R.F.S.) using the same criteria. DNA was extracted from saliva according to a published protocol (Deeley et al., 2016) and samples were sequenced in both directions to determine the presence of the *SCN8A* Met136Val mutation in the exon 4 of the gene. We designed a set of primers (5ʹ TGT GCT TCA TCT CCT TTC AGG 3ʹ forward and 5ʹ CCA CAT TCT TCG ACC AGT CA 3ʹ reverse) using the Primer3 online software (http://primer3.ut.ee/). Polymerase chain reactions conditions were 30 cycles at 95°C for 30 seconds, 55°C for 30 seconds, and 72°C for 1 minute, followed by a 7‐minute hold at 72°C. All samples were sequence in both directions. Sequences were analyzed using the DNA Analysis Software Sequencher 5.0 (Sequencher 5.0: Gene Codes; http://genecodes.com/).

We did not find any case with the *SCN8A* Met136Val, which suggests that this mutation is an infrequent cause of cTN.

These findings should help guide future study designs that aim to identify the associations between sodium channel genes and trigeminal neuralgia. Analyses of common variants of Na_v_1.7 (*SCN9A* rs6746030) and nerve growth factor receptor (*NTRK1* rs633) of 48 individuals did not show overrepresentation of alleles in cases with trigeminal neuralgia (Costa et al., 2019) and it is reasonable to propose that future studies should instead focus on detecting rare variants that may each account for small fractions of the population of patients with cTN (Di Stefano et al., 2020).

## ETHICAL COMPLIANCE

This study was approved by the University of Pittsburgh Institutional Review Board.

## CONFLICT OF INTEREST

The authors have no conflict of interest to declare.

## AUTHORS’ CONTRIBUTION

Raymond F. Sekula: Proposed the concept, designed the study, collected and interpreted the data, obtained support, and critically revised the manuscript. Kathleen Deeley: Collected, generated, and analyzed the data, and critically revised the manuscript. Hayley Denwood: Generated data and critically revised the manuscript. Alexandre R. Vieira: Proposed the concept, designed the study, analyzed and interpreted the data, obtained support, and wrote the manuscript.
